# Supplementary material for: Preparatory phase of large earthquakes illuminated by unsupervised categorization of earthquake catalog features
Source: Nat Commun. 2026 May 4;17:4024. doi: 10.1038/s41467-026-72279-x (PMC13139499; doi:10.1038/s41467-026-72279-x)
Supplement: Supplementary file 1 — Supplementary information [file 41467_2026_72279_MOESM1_ESM.pdf]

Supplementary information

Preparatory phase of large earthquakes illuminated by unsupervised categorization of earthquake catalog features

Sadegh Karimpouli[1*], Patricia Martínez-Garzón[1,2], Sebastián Núñez-Jara[1], Matteo Picozzi[3], Daniele Spallarossa[4], Grzegorz Kwiatek[5], Georg Dresen[1,6], Marco Bohnhoff[1,7], Gregory C. Beroza[8]

1. GFZ Helmholtz Center for Geosciences, Potsdam, Germany.
2. RWTH University of Aachen, Aachen, Germany
3. National Institute of Oceanography and Applied Geophysics – OGS, Trieste, Italy.
4. DISTAV, University of Genoa, Genoa, Italy.
5. GMuG Gesellschaft für Materialprüfung und Geophysik mbH, Dieselstraße 9, D-61231 Bad Nauheim, Germany.
6. University of Potsdam, Potsdam, Germany
7. Department of Earth Sciences, Free University Berlin, Berlin, Germany.
8. Department of Geophysics, Stanford University, Stanford, CA, USA.

* Corresponding author: sadegh.karimpouli@gfz.de

This supplementary material provides additional details supporting the findings presented in the main manuscript. Tables summarize the features, time and space windows, and computational parameters employed in the study, while figures illustrate the spatial and temporal distribution of seismic families, feature evolution, and clustering results for the analysed earthquake sequences. All data and codes used in this study are made available for reproducibility and further research.

Table S1. Features summary.

	Feature type (Reference)	Feature name	Notation	Description
1	(Scholz 1968) [1]	Event rate	n	Number of events in a time interval.
2	(Gutenberg & Richter 1944) [2]	b-value	bp	The slope of a Gutenberg–Richter frequency-magnitude distribution. Here we use b-positive method by van der Elst (2021).
3	Localization in space (Kagan & Knopoff 1980) [3]	Correlation integral	C	A measure of the spatial correlation of seismicity location, defined as the fraction of event pairs within a given distance.
4	Localization in time	Interevent time	IEt	Time difference between two subsequent events

5	Localization in space	Interevent distance	IEs	Spatial difference between hypocenters of two subsequent events.
6	Clustering feature (Localization in space-time-magnitude) (Zaliapin and Ben-Zion 2013; Martınez-Garzon et al. 2019) [4,5]	Product of T and R	trp	Product of normalized time and space components of nearest-neighbor space-time-magnitude distances
7		Ratio of T and R	trr	Ratio of normalized time and space components of nearest-neighbor space-time-magnitude distances
8		Proportion of foreshocks	pfo	Proportion of foreshock events computed based on clustering analysis.
9		Proportion of mainshocks	pma	Proportion of mainshock events computed based on clustering analysis.
10		Proportion of aftershocks	paf	Proportion of aftershock events computed based on clustering analysis.
11		Clustered events ratio	cer	Ratio of clustered events to all events.
12	Localisation in space	3D convex Hull volume	v	The volume enclosed by the smallest convex polyhedron that fully contains the given set of locations of events in three-dimensional space.
13	(Picozzi et al. 2022) [6]	Energy index	Ei	The difference between the observed energy and the values associated with the median empirical scaling model.
14		Moment rate	logM	Logarithm of cumulative seismic moment in a time interval.
15	(Kostrov 1974) [7]	Kostrov strain	logKs	Logarithm of cumulative Kostrov strain.
16	Family features (Zaliapin and Ben-Zion 2013; Karimpouli et al. 2024) [8,9]	Number of families	num	Number of families in a time interval.
17		Family rate	mem	Average of family members (events) in a time interval.
18		Family members	num_nodes	Number of members (events) in the family.
19		Family connections	num_edges	Number of connections between members of a family.

20		Family length	len<br>**time-len**	Average temporal length of families (time difference between first and last family members)
21		Family size	siz<br>**spatial_siz**	Average spatial size of families (longest distance among family members)
22		Family radius	rad<br>**radius**	Eccentricity of a family member is the longest shortest path from a member to any other member. Family radius is the minimum eccentricity of all members in the family.
23		Family diameter	dim<br>**diameter**	Eccentricity of a family member is the longest shortest path from a member to any other member. Family diameter is the maximum eccentricity of all members in the family.
24		Family density	den<br>**density**	A measure of the number of connections in the family relative to the maximum possible number of connections.

The features with regular fonts are computed via the event-based method. The features with **bold** fonts are computed per-family as topological features.

Table S2. Time and space windows.

	Event- and family-based	
Case	Time window (day)	Space window (km)
Kahramanmaraş	15, 30	17, 33
L'Aquila	15, 30	10, 20
Iquique	15, 30	17, 33
Amatrice	15, 30	10, 20
Noto	15, 30	10, 20

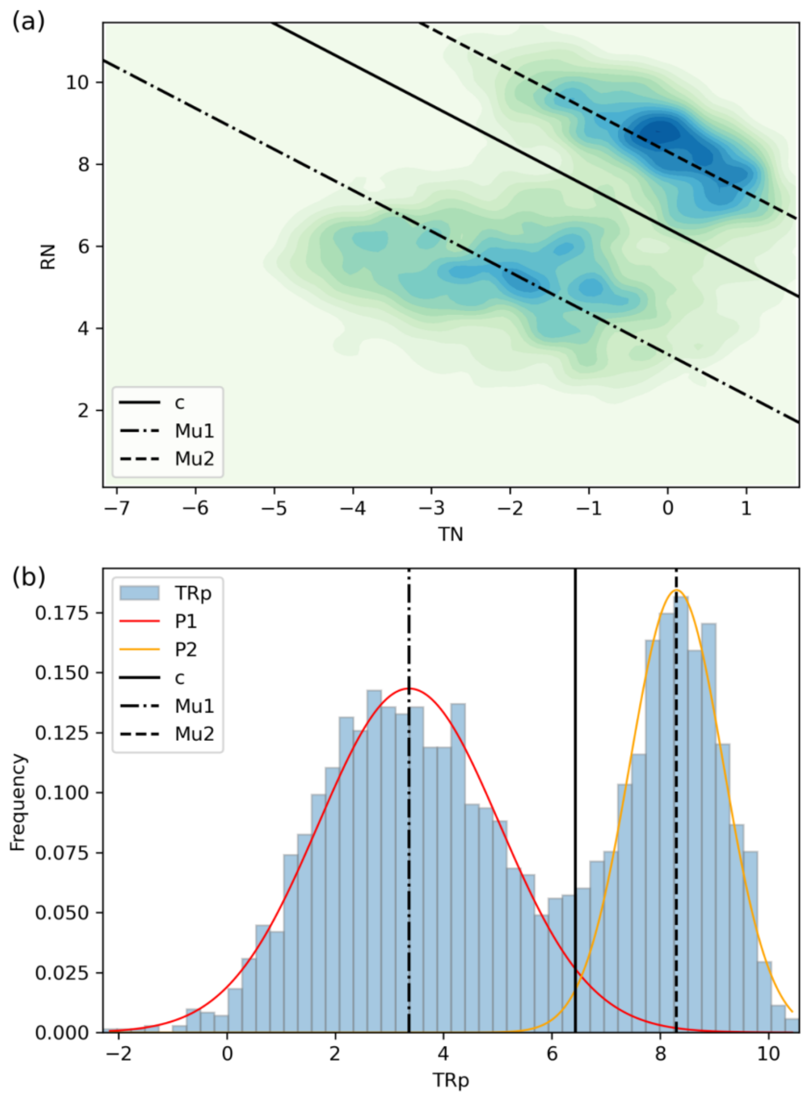

Fig. S1. Distribution of (a) the normalized time (TN) and space (RN) components and (b) the nearest neighbor distance (TRp) for the seismicity before the Kahramanmaraş earthquake. The Gaussian mixture model is fitted with two components to separate background and clustered populations (P1, P2) with mean values of Mu1 and Mu2, where c is the separation value (or threshold).

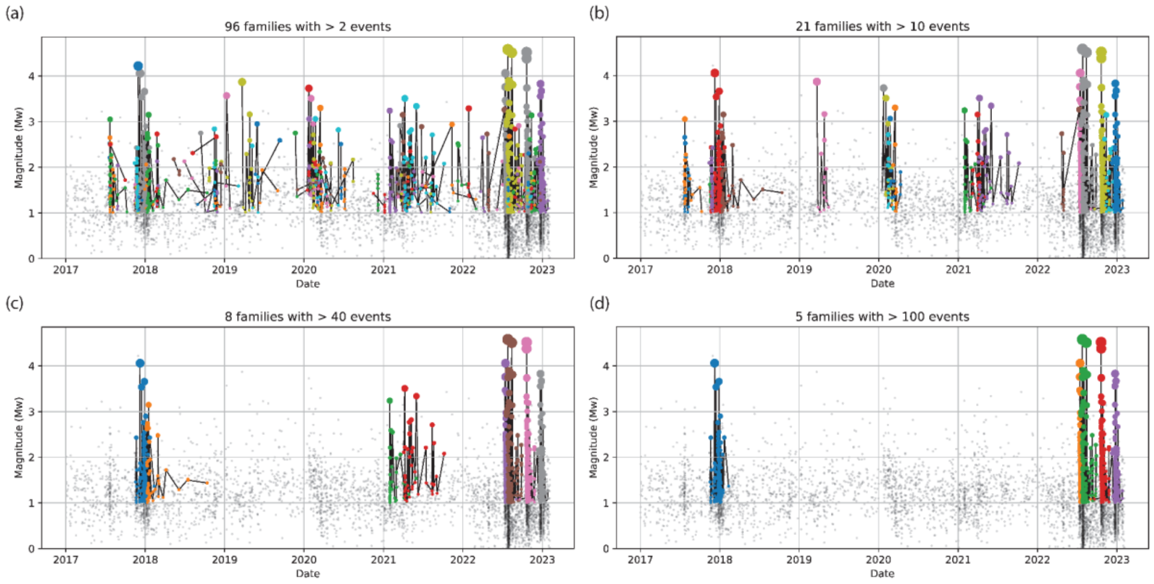

Fig. S2. Seismicity families of Kahramanmaraş earthquake with a number of more than (a) 2, (b) 10, (c) 40, and (d) 100 events. Colors are arbitrarily selected to show connected members of a family. These families represent the clustered seismicity after the removal of single background events.

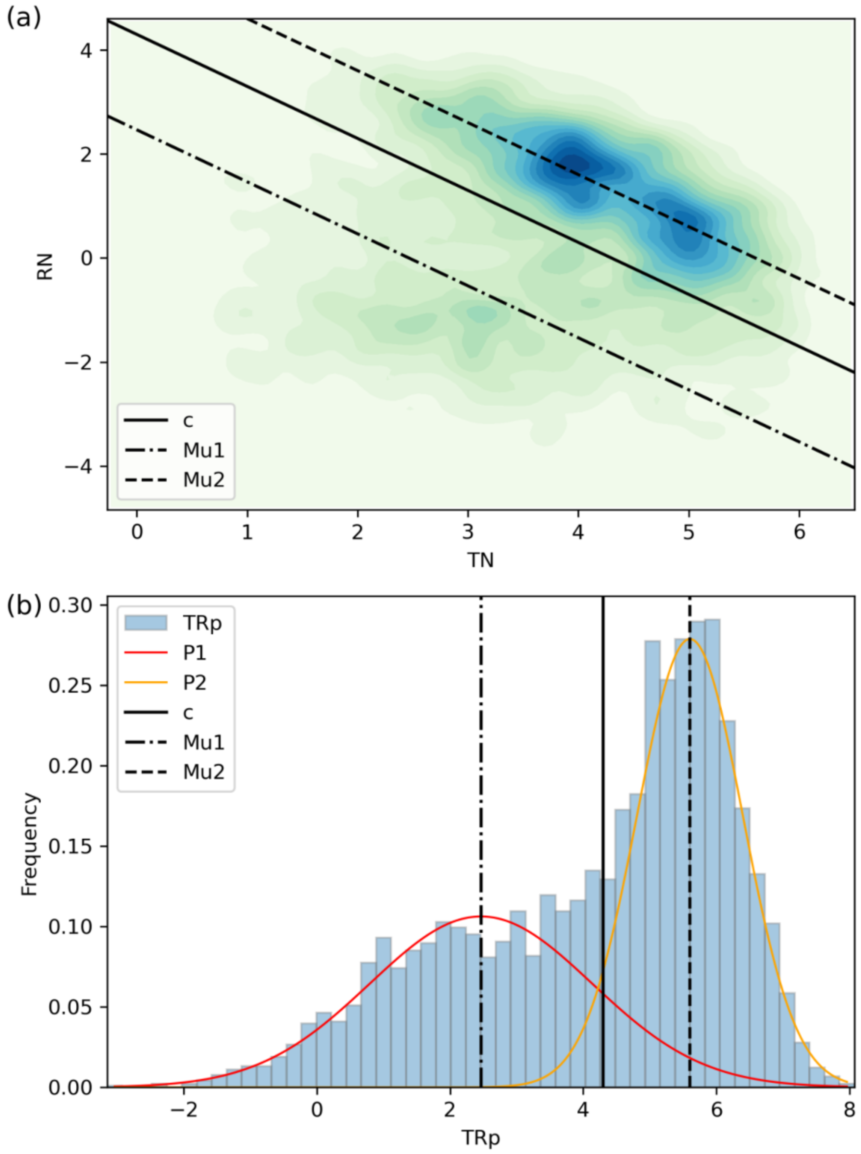

Fig. S3. Distribution of (a) the normalized time (TN) and space (RN) components and (b) the nearest neighbor distance (TRp) for the seismicity before the L'Aquila earthquake. The Gaussian mixture model is fitted with two components to separate background and clustered populations (P1, P2) with mean values of Mu1 and Mu2, where c is the separation value (or threshold).

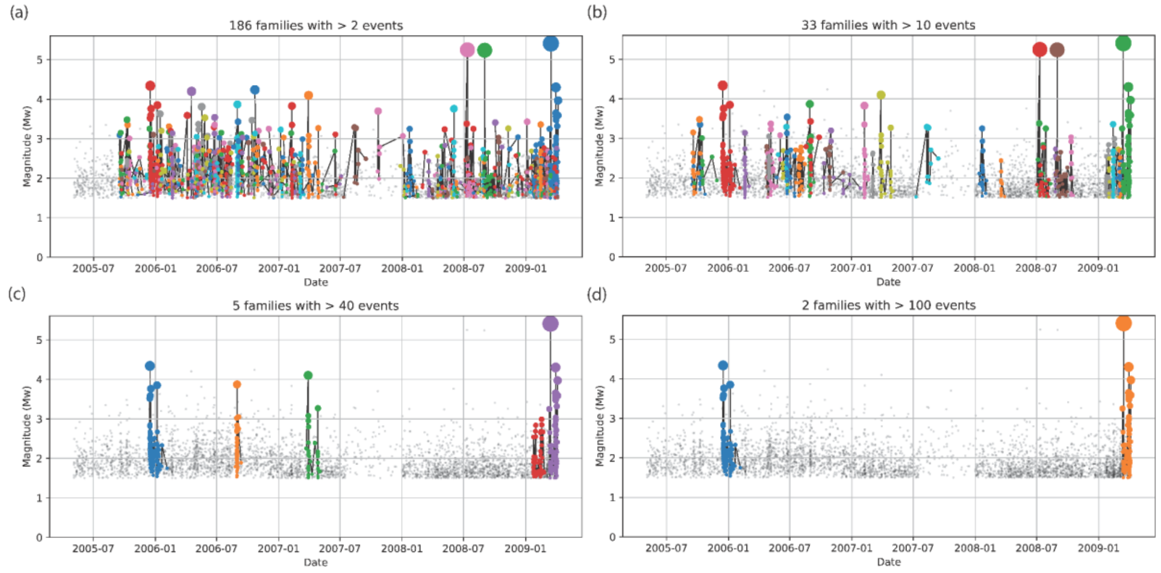

Fig. S4. Families of L'Aquila earthquake with number of more than (a) 2, (b) 10, (c) 40, and (d) 100 events. Colors are arbitrarily selected to show connected members of a family.

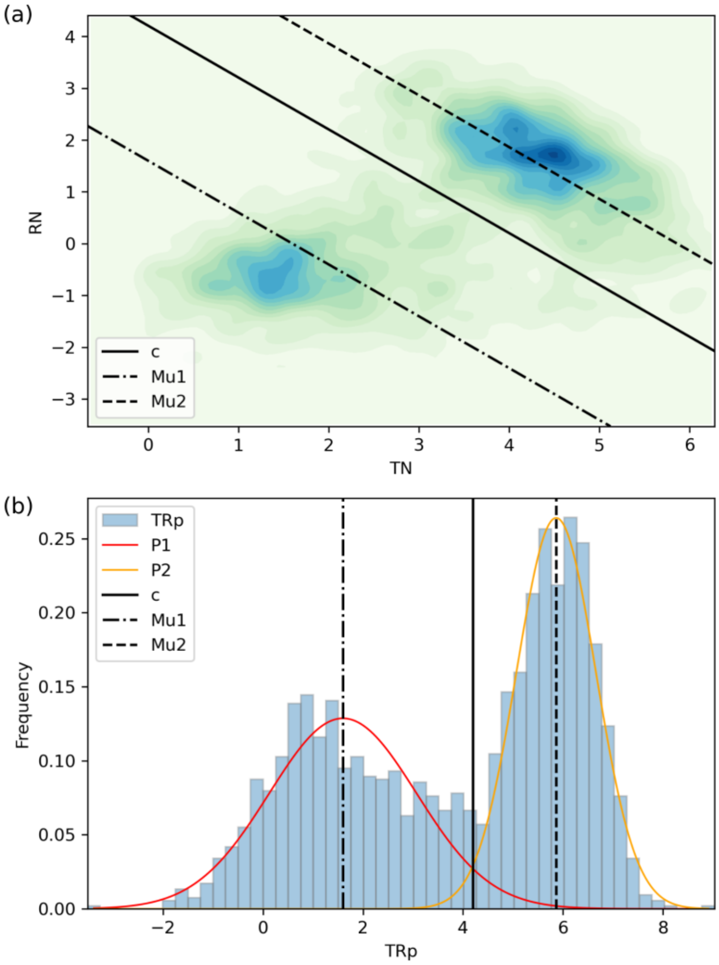

Fig. S5. Distribution of (a) the normalized time (TN) and space (RN) components and (b) the nearest neighbor distance (TRp) for the seismicity before the Iquique earthquake. The Gaussian mixture model is fitted with two components to separate background and clustered populations (P1, P2) with mean values of Mu1 and Mu2, where c is the separation value (or threshold).

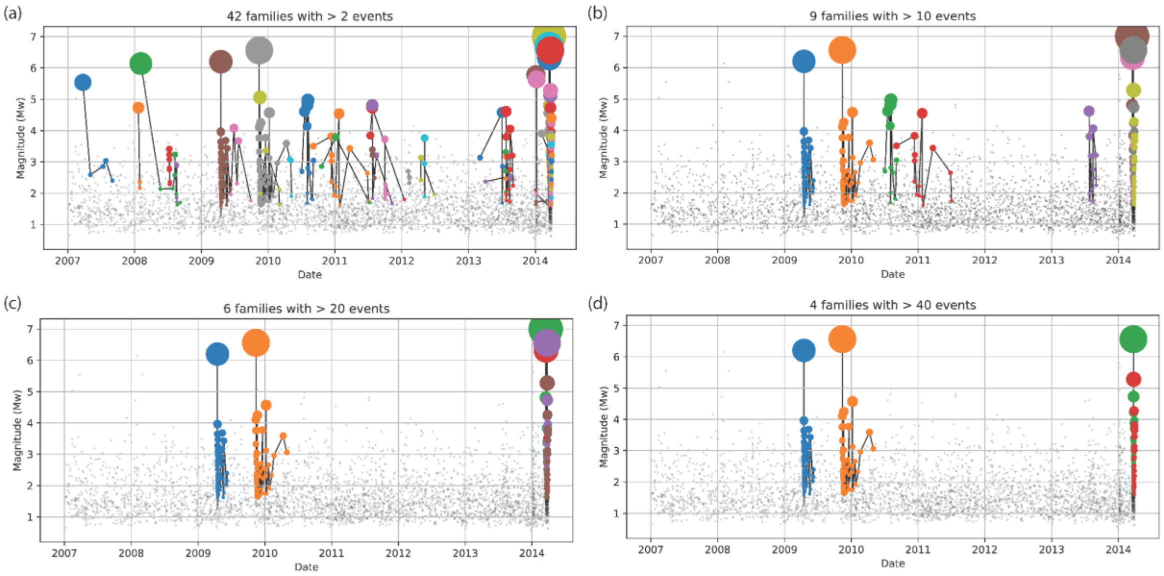

Fig. S6. Families of Iquique earthquake with number of more than (a) 2, (b) 10, (c) 20, and (d) 40 events. Colors are arbitrarily selected to show connected members of a family.

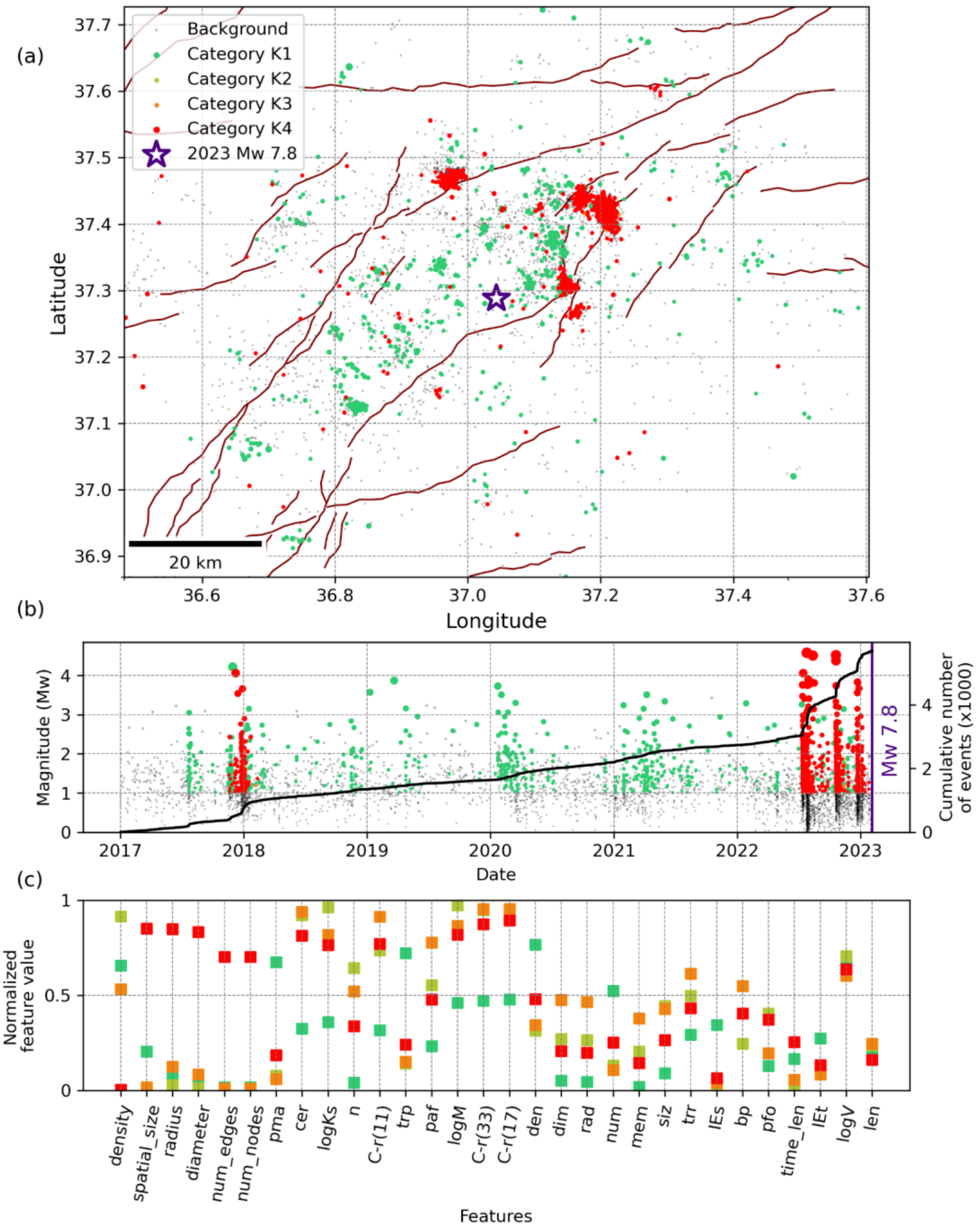

Fig. S7. Categorization of seismicity families prior to the 2023 M_W 7.8 Kahramanmaraş using **Spectral clustering** algorithm. Results show that 3 categories (K2-K4) are found in the preparatory phase of the 2023 mainshock and seismicity localization in 2018, while other families are accounted as one similar category (K1). This shows that this algorithm is not able to produce more details for less critical families.

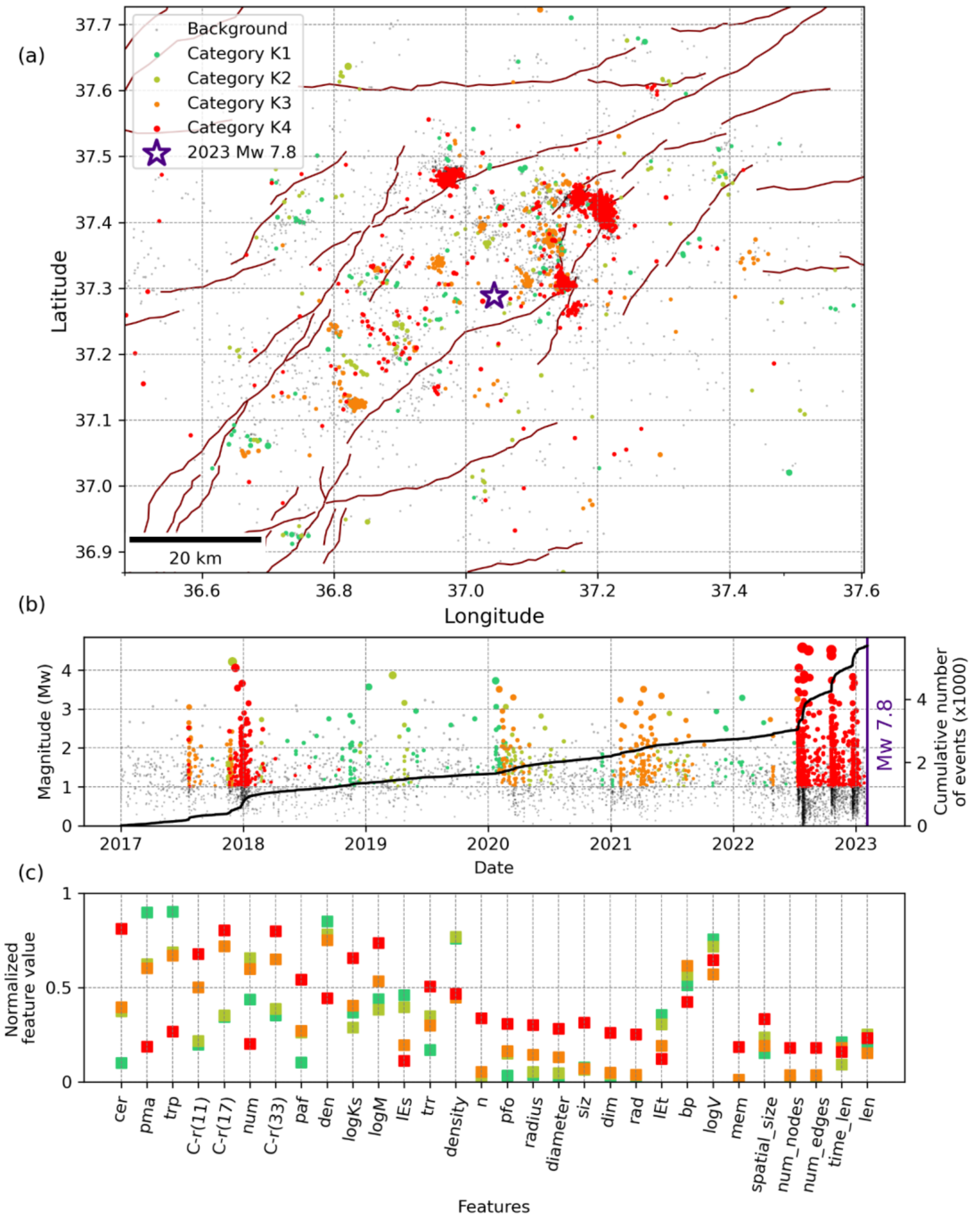

Fig. S8. Categorization of seismicity families prior to the 2023 M_W 7.8 Kahramanmaraş using **'Ward' hierarchical clustering** algorithm. Compared to the K-means algorithm (Fig. 3), this algorithm separates less critical families more than more critical ones. For example, seismicity localization in 2018 is categorized similar to the preparatory phase of the 2023 mainshock (K4), while only the later led to a mainshock.

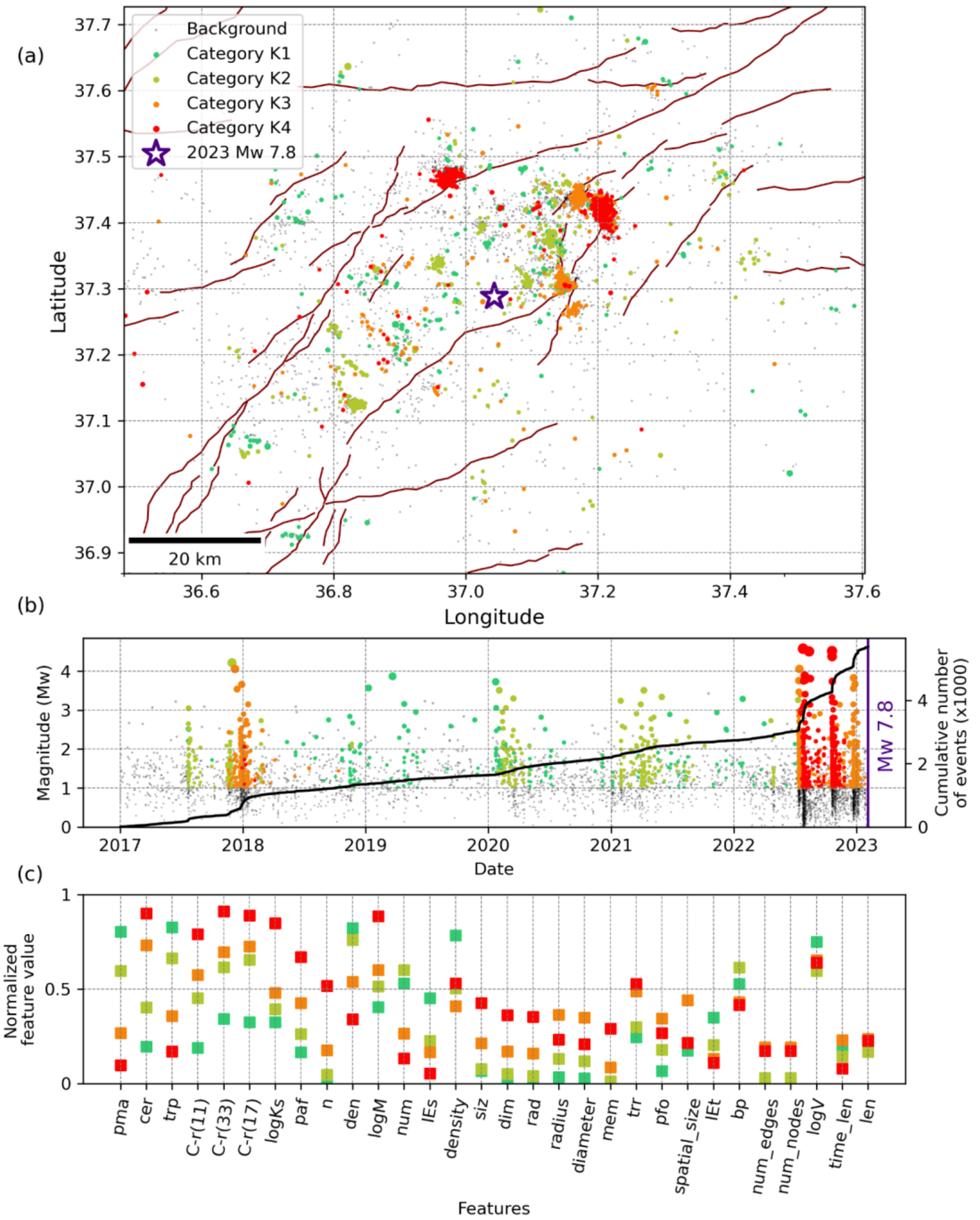

Fig. S9. Categorization of seismicity families prior to the 2023 M_W 7.8 Kahramanmaraş using Gaussian Mixture Model (GMM) algorithm. These results are very similar to the results obtained by the K-means algorithm.

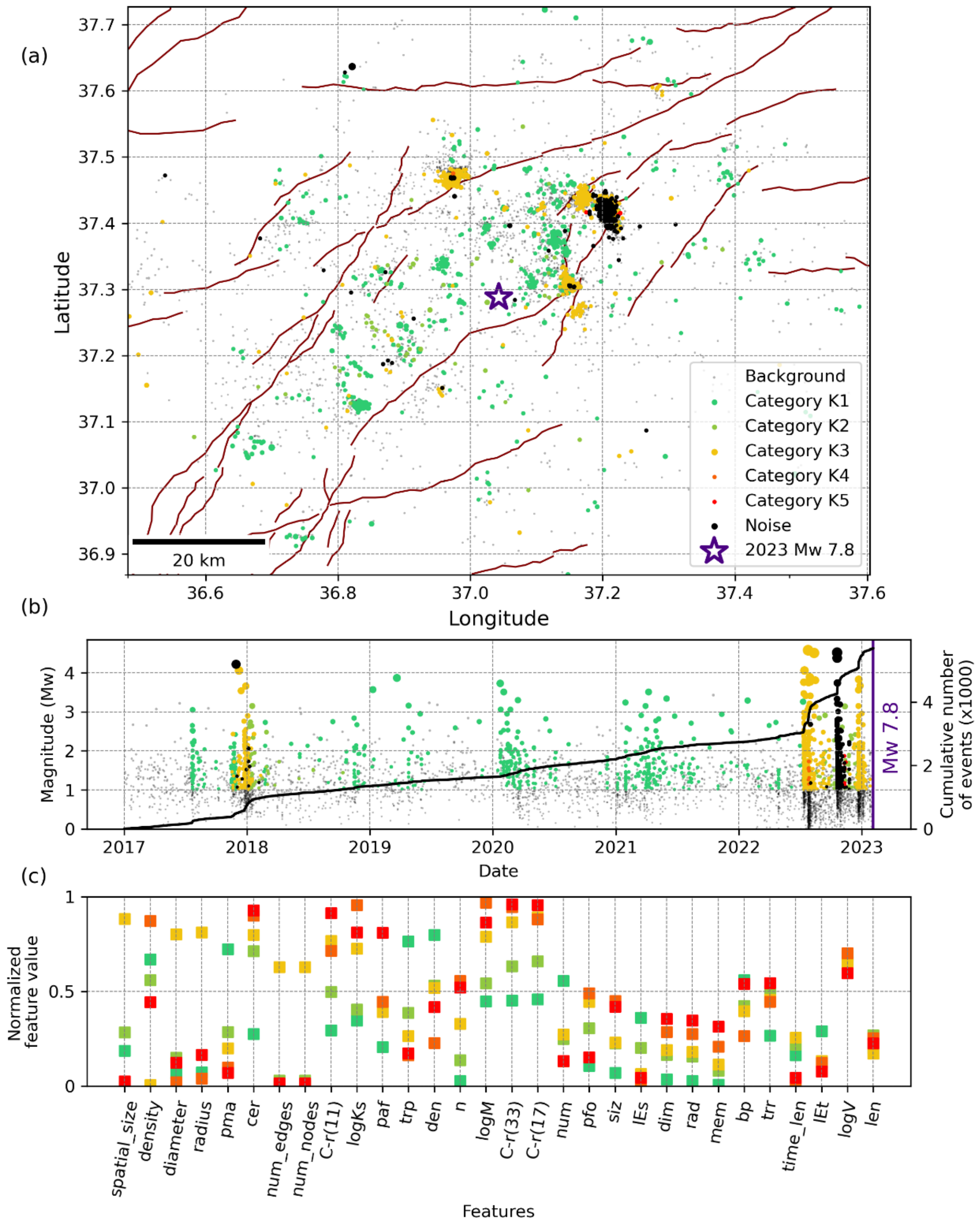

Fig. S10. Categorization of seismicity families prior to the 2023 M_W 7.8 Kahramanmaraş using **Density-Based Spatial Clustering of Applications with Noise (DBSCAN)** algorithm. As it is illustrated, DBSCAN mostly separates the families based on their topological features, where a critical family is assumed as noise to other families. We searched in a grid to find the optimum values for epsilon (maximum distance between two samples) and minimum number of samples in a neighbourhood for a family to be considered as a core of a cluster.

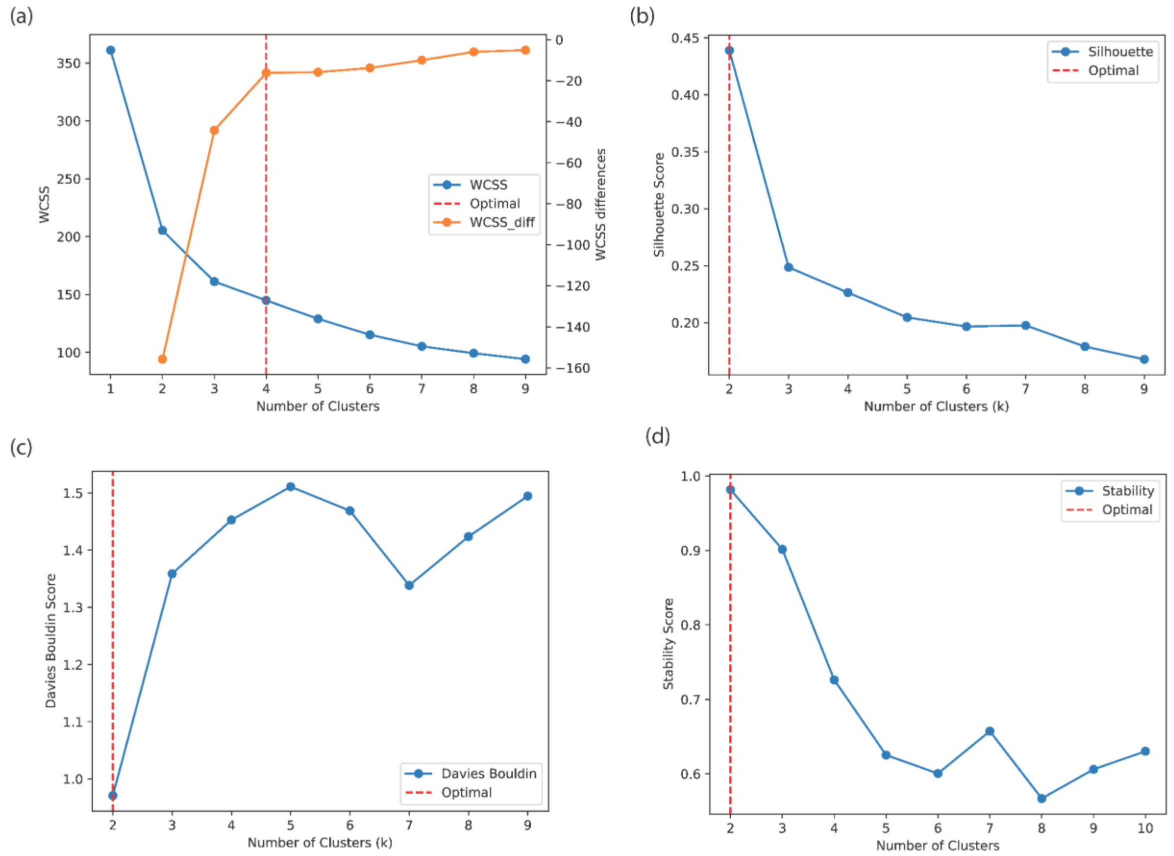

Fig. S11. Quantitative metrics for detecting number of categories (clusters) with (a) Within Category Sum of Square (WCSS) distance (elbow point is optimum), (b) silhouette score (the higher the better), (c) Davies-Bouldin score (lower is better) and (d) Stability criterion (the higher the better).

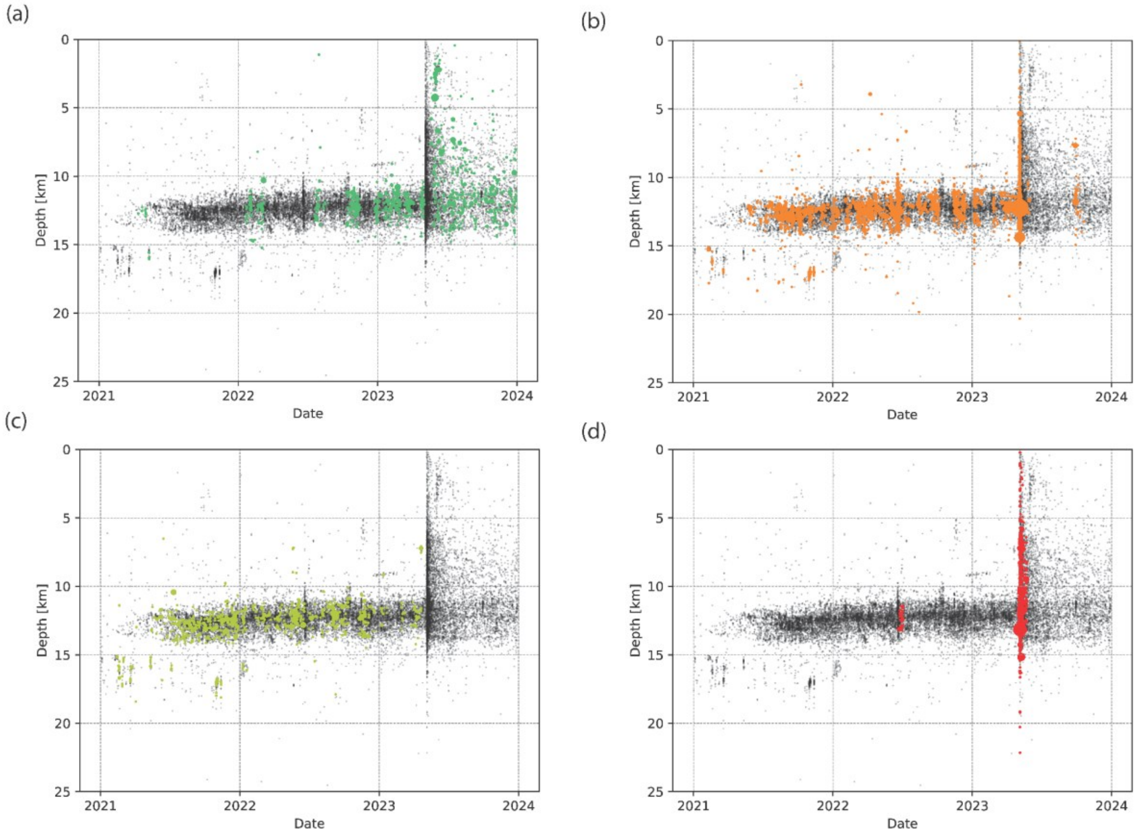

Fig. S12. Depth distribution of four categories (a-d) in the 2024 M_W 7.5 Noto earthquake. The 2023 M_W 6.2 event shows up as the most critical category (d). The seismicity patterns change completely before (b, c) and after (d) this event, which reactivates the Suzu Blind Fault (SBF) and causing a new seismicity pattern separated from shallow to deep depths.

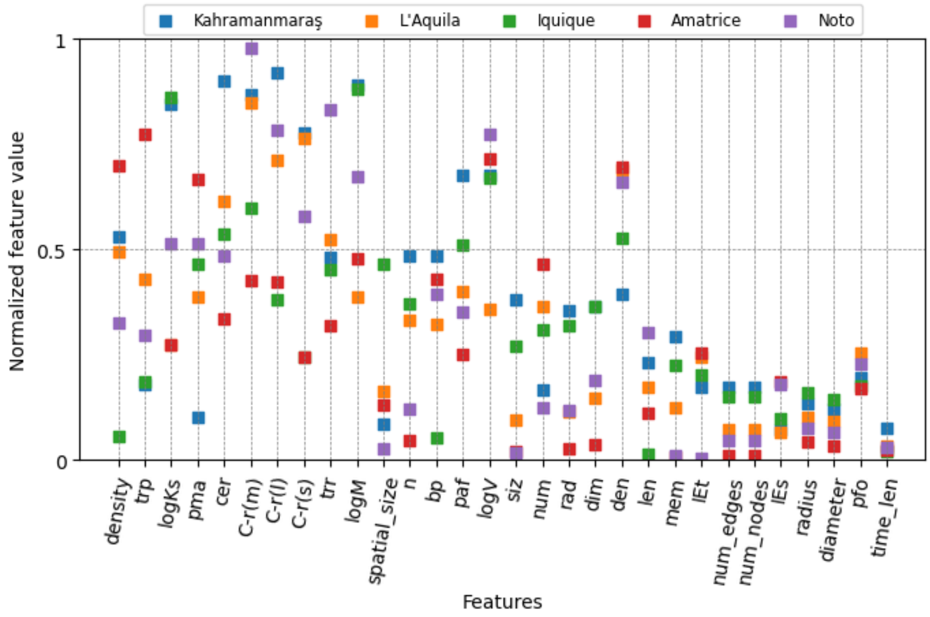

Fig. S13. Comparison of the features values in category centroids for all cases. For three cases with known preparatory phases, i.e., Kahramanmaraş, L'Aquila and Iquique, we selected the most critical categories (K4, L5, and I4). We selected A3 for the Amatrice (as the category before the mainshock) and N3 for the Noto case (as the main swarm-type category).

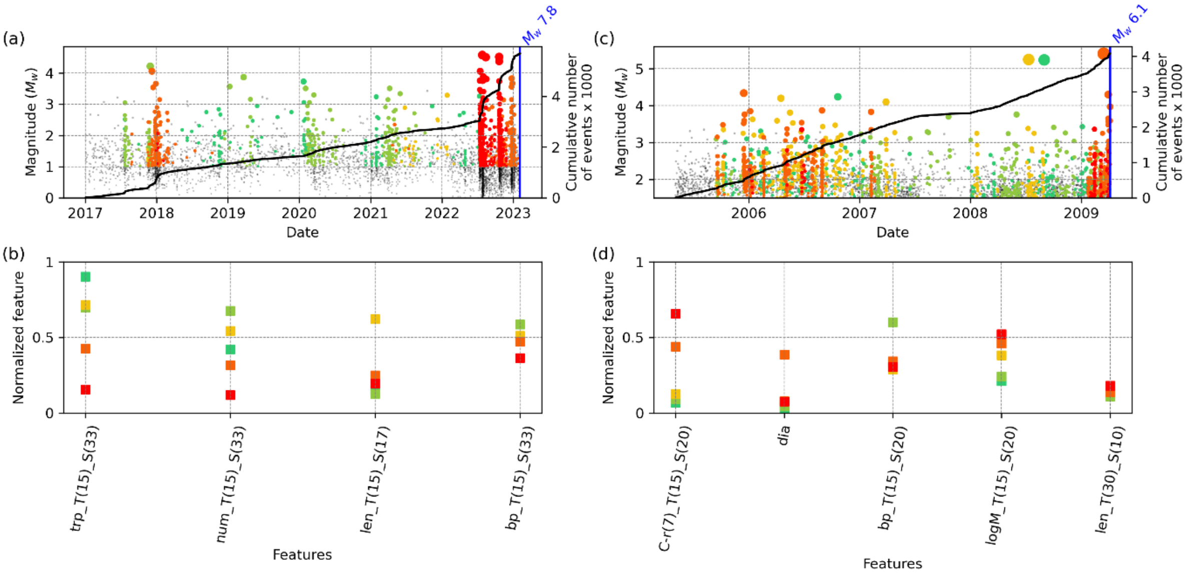

Fig. S14. Results from categories of seismicity families based on **the most independent features** prior to the 2023 M_w 7.8 Kahramanmaraş and 2009 M_w 6.1 L'Aquila earthquakes. (a, c) Magnitude-time distribution of event family members, with the cumulative number of (all background and clustered) events represented by solid lines. (b, d) Feature values at the centroid of each category, sorted from highest to lowest separability. The color scheme reflects the evolution of families, transitioning from a stable state (green) to a critical state (red). Description and explanations of individual features is provided in Table S1. 'T' and 'S' are time and space windows in 'day' and 'km'.

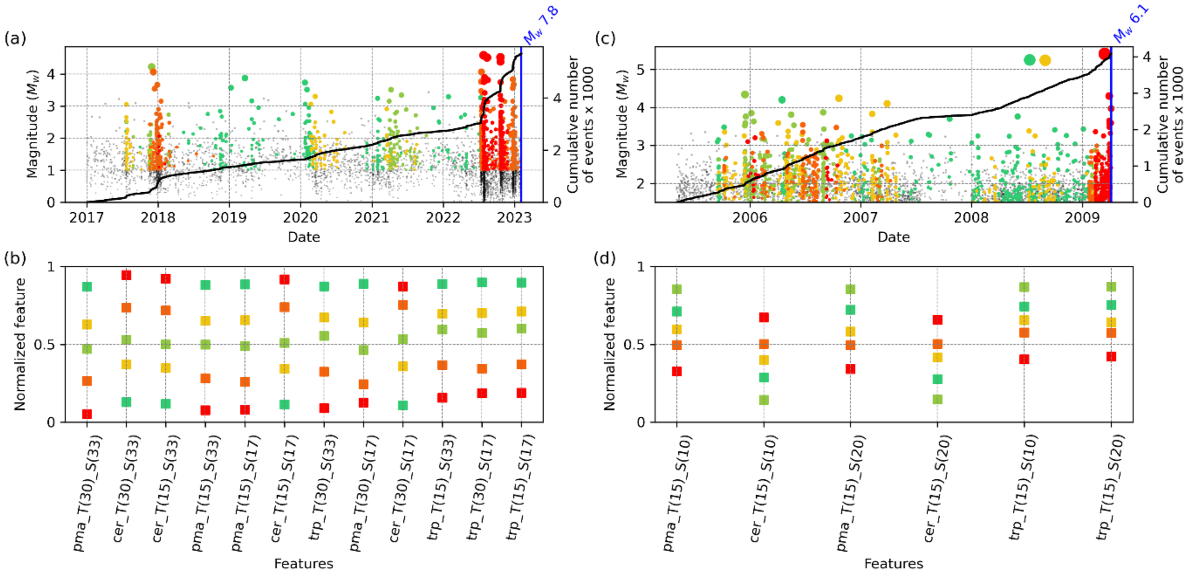

Fig. S15. Results from categories of seismicity families based on **the most correlated features** prior to the 2023 M_W 7.8 Kahramanmaraş and 2009 M_W 6.1 L'Aquila earthquakes. (a, c) Magnitude-time distribution of event family members, with the cumulative number of (all background and clustered) events represented by solid lines. (b, d) Feature values at the centroid of each category, sorted from highest to lowest separability. The color scheme reflects the evolution of families, transitioning from a stable state (green) to a critical state (red). Description and explanations of individual features is provided in Table S1. 'T' and 'S' are time and space windows in 'day' and 'km'.

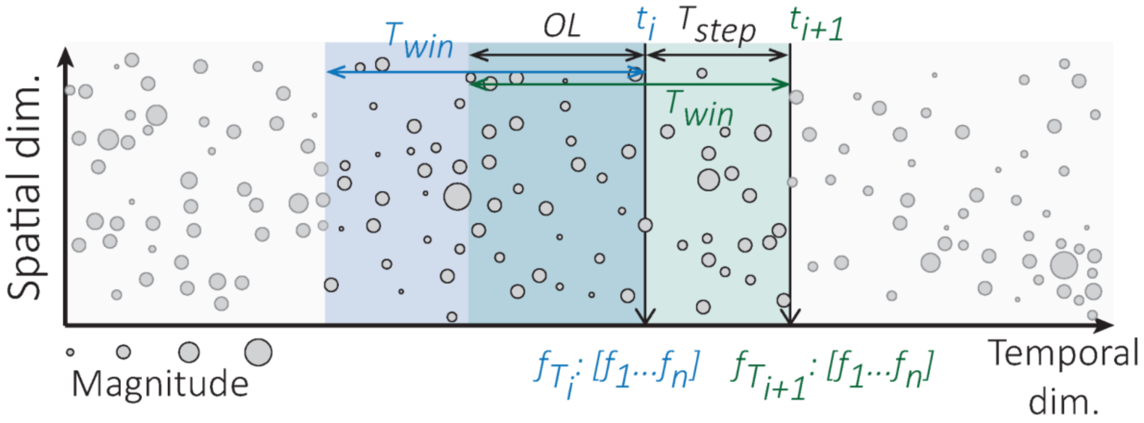

Fig. S16. Schematic illustration of time-based feature computation.

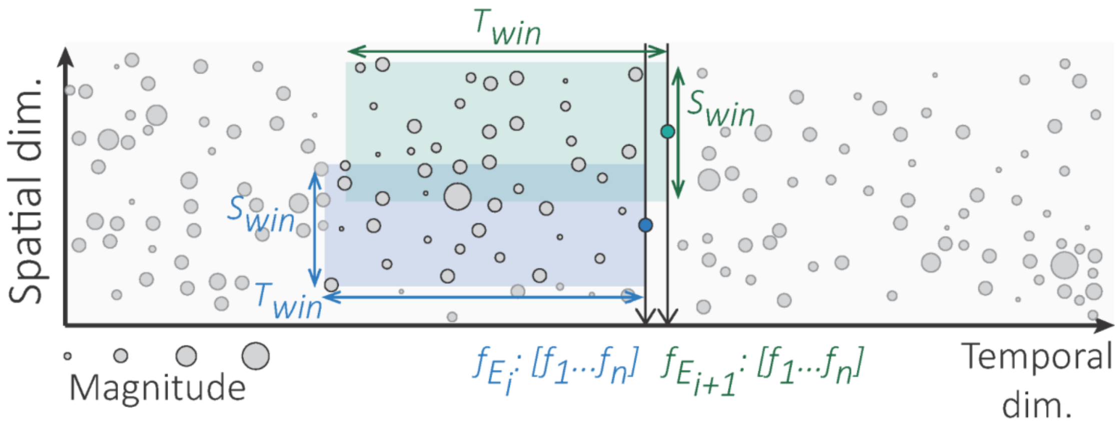

Fig. S17. Schematic illustration of event-based feature computation.

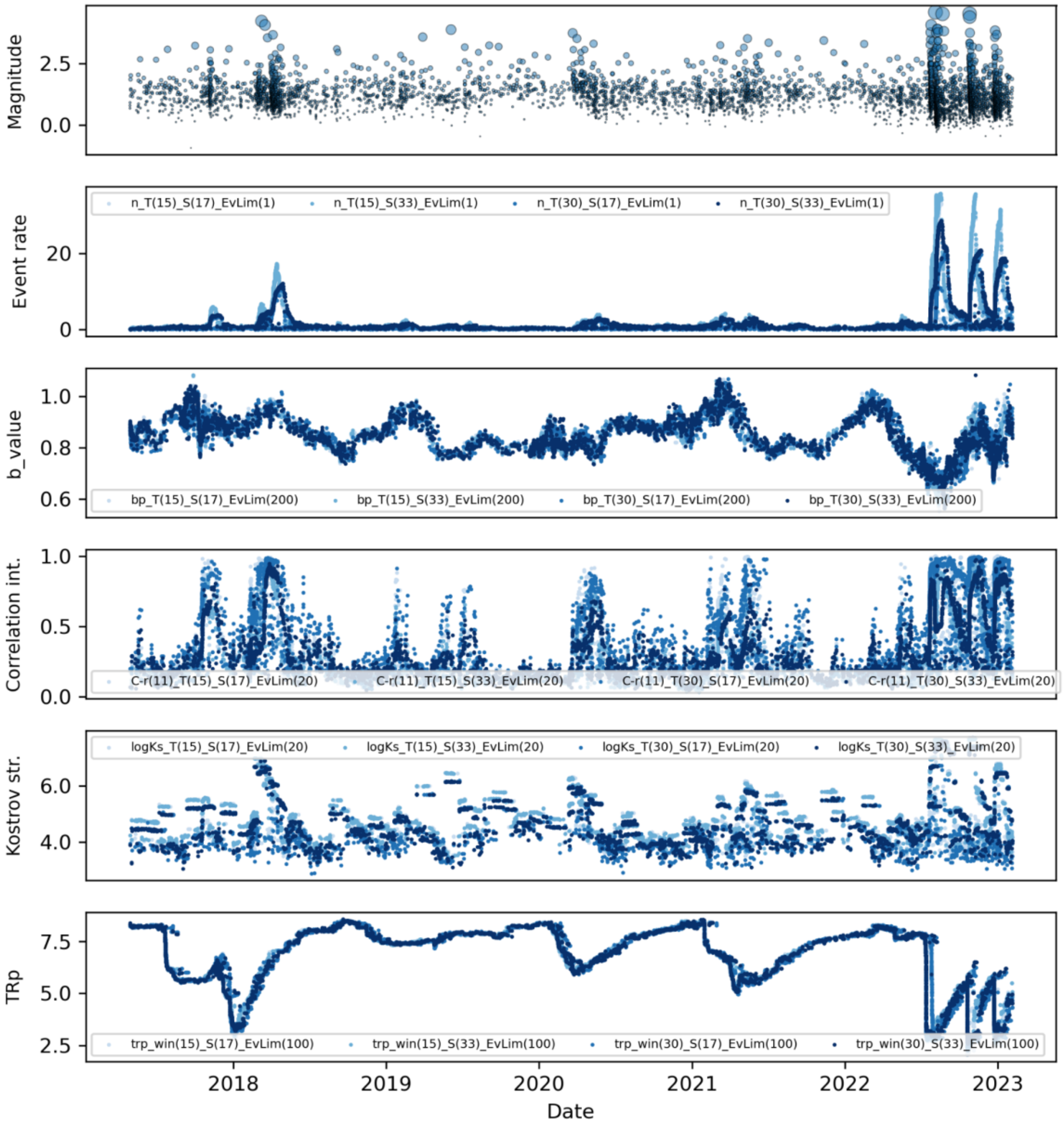

Fig. S18. Event-based features computed for Kahramanmaraş earthquake. For features notation see Table S1. 'T' is the time window [day], 'S' is the space window [km] and 'EvLim' is the minimum number of events used for computation.

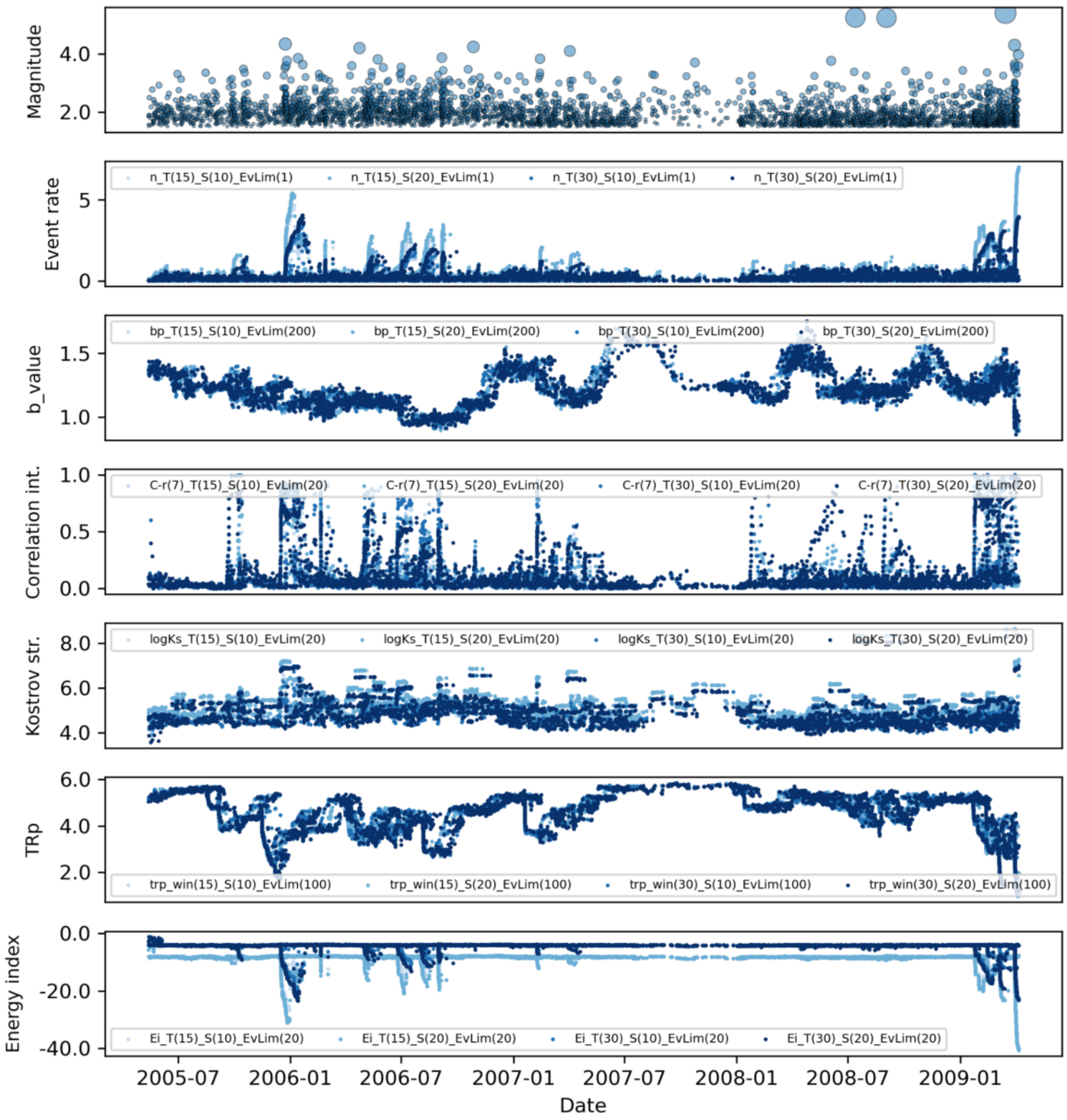

Fig. S19. Event-based features computed for L'Aquila earthquake. For features notation see Table S1. 'T' is the time window [day], 'S' is the space window [km] and 'EvLim' is the minimum number of events used for computation.

References

1 Scholz, C. H. Experimental study of the fracturing process in brittle rock. *Journal of Geophysical Research* **73**, 1447–1454 (1968). https://doi.org/10.1029/JB073I004P01447

2 Gutenberg, B. & Richter, C. F. Frequency of earthquakes in California*. *Bulletin of the Seismological Society of America* **34**, 185–188 (1944). https://doi.org/10.1785/BSSA0340040185

3 Kagan, Y. Y. & Knopoff, L. Spatial distribution of earthquakes: the two-point correlation function. *Geophysical Journal International* **62**, 303–320 (1980). https://doi.org/10.1111/J.1365-246X.1980.TB04857.X

4 Zaliapin, I. & Ben-Zion, Y. Earthquake clusters in southern California II: Classification and relation to physical properties of the crust. *Journal of Geophysical Research: Solid Earth* **118**, 2865–2877 (2013). https://doi.org/10.1002/JGRB.50178

5 Martínez-Garzón, P., Ben-Zion, Y., Zaliapin, I. & Bohnhoff, M. Seismic clustering in the Sea of Marmara: Implications for monitoring earthquake processes. *Tectonophysics* **768**, 228176–228176 (2019). https://doi.org/10.1016/J.TECTO.2019.228176

6 Picozzi, M., Spallarossa, D., Bindi, D., Iaccarino, A. G. & Rivalta, E. Detection of Spatial and Temporal Stress Changes During the 2016 Central Italy Seismic Sequence by Monitoring the Evolution of the Energy Index. *Journal of Geophysical Research: Solid Earth* **127**, e2022JB025100–e022022JB025100 (2022). https://doi.org/10.1029/2022JB025100

7 Kostrov V, V. Seismic moment and enengy of earthquakes, and the seismic flow of rock, Izv. Acad. Sci. USSR. *Phys. Solid Earth* **1**, 23–44 (1974).

8 Zaliapin, I. & Ben-Zion, Y. Earthquake clusters in southern California I: Identification and stability. *Journal of Geophysical Research: Solid Earth* **118**, 2847–2864 (2013). https://doi.org/10.1002/JGRB.50179

9 Karimpouli, S. *et al.* Empowering Machine Learning Forecasting of Labquake Using Event-Based Features and Clustering Characteristics. *Journal of Geophysical Research: Machine Learning and Computation* **1**, e2024JH000160–e002024JH000160 (2024). https://doi.org/10.1029/2024JH000160
